# Demonstration of the Optical Isotropy of TiO_2_ Thin Films Prepared by the Sol–Gel Method

**DOI:** 10.3390/ma17143391

**Published:** 2024-07-09

**Authors:** Jacek Nizioł, Magdalena Zięba, Maciej Śniechowski, Ewa Gondek, Wojciech Pakieła, Paweł Karasiński

**Affiliations:** 1 Faculty of Physics and Applied Computer Science, AGH University of Kraków, al. Mickiewicza 30, 30-059 Krakow, Poland; maciej.sniechowski@fis.agh.edu.pl; 2 Department of Optoelectronics, Silesian University of Technology, ul. B. Krzywoustego 2, 44-100 Gliwice, Poland; magdalena.zieba@polsl.pl (M.Z.); pawel.karasinski@polsl.pl (P.K.); 3 Institute of Physics, Cracow University of Technology, ul. Podchorążych 1, 30-084 Kraków, Poland; ewa.gondek@pk.edu.pl; 4 Department of Engineering Materials and Biomaterials, Silesian University of Technology, ul. Konarskiego 18a, 44-100 Gliwice, Poland; wojciech.pakiela@polsl.pl

**Keywords:** titanium dioxide, spectroscopic ellipsometry, thin layers, sol–gel, planar optical waveguides

## Abstract

Titanium dioxide (TiO_2_) thin films prepared by the sol–gel technique have been shown to be optically isotropic and, unlike the films obtained by competitive methods, do not exhibit measurable birefringence. A series of submicrometer-thin titanium dioxide films were prepared using the sol–gel technique and then thermally annealed at different temperatures. The samples were analyzed by spectroscopic ellipsometry using the Mueller matrix formalism, X-ray diffractometry and scanning electron microscopy. The conversion of amorphous titanium dioxide to polycrystalline anatase occurred at 400 °C or higher. Crystallites of a few percent of the film thickness were observed. Nevertheless, the crystallization process did not trigger the appearance of birefringence. These observations demonstrate that high-quality planar optical waveguides can be successfully fabricated on flexible substrates, in particular those composed of efficient polymers that can withstand the aforementioned temperatures.

## 1. Introduction

Titanium dioxide TiO_2_ (also known as titania) naturally exists in three polymorphs, namely anatase (tetragonal), rutile (tetragonal) and brookite (orthorhombic—rare if compared to anatase and rutile) [1]. Thermodynamically, the rutile structure is the stable one. Moreover, more than ten other polymorphs have been obtained in the laboratory under controlled high-pressure conditions [2].

The wide availability (and therefore low price) of this ceramic material, combined with its chemical stability, biocompatibility and non-toxicity, make it commercially important for applications in various fields. The most common application is white pigment in paints, printing inks, plastics, ceramics and cosmetics [3]. Other promising areas of practical implementation include, for example, solar cells [4], self-cleaning coatings on surfaces [5], photocatalytic applications [6], gas sensors [7] or nanobiology and nanomedicine [8]. A more comprehensive characterization of this material and a discussion of its future prospects can easily be found in the dedicated monographs or review papers [9,10,11,12,13]. Efficient photonic integrated circuits (PICs) operating at typical telecommunication windows are becoming increasingly demanded in many applications ranging from ultra-dense data processing and quantum technologies [14,15] to biomedical sensing [16,17]. Planar waveguides are an integral part of integrated optics. They evolve rapidly because the ultimate objective is to integrate the optical circuits with electronic systems on a single silicon chip. Such structures will revolutionize the electronics industry in terms of increasing the operational speed. To achieve the objectives mentioned above, compactness and low losses are two basic requirements that can be met by the use of titanium dioxide.

TiO_2_ crystals have high resistivity (~10^15^ Ω·cm) [18]. Oxygen vacancies, titanium interstitials and reduced crystal surfaces introduce shallow electron donor energy states that enhance the electrical conductivity of titanium dioxide [19] and thus define the n-type character of TiO_2_. However, this wide-bandgap semiconductor can be switched to p-type by ion implantation [20]. One should add to the above the lack of significant absorption in the vis-NIR optical wavelength range [21], a band edge located near 400 nm combined with a high refractive index (>2.5 at the anatase phase and >2.7 at the rutile phase) [10] and, finally, a rather low thermal expansion coefficient up to 1000 °C. All these properties make titanium dioxide appear to be virtually the ideal material for PIC applications. Unfortunately, the preparation of TiO_2_ in the form of thin (submicrometer) layers is challenging on some substrates. Commonly, titanium dioxide is deposited through a variety of sputtering processes or physical vapor deposition [22,23,24,25,26]. However, such methods are constrained in the case of deposition on thermally unstable substrates, i.e., polymers. These are materials characterized by a relatively low glass transition and melting temperatures, so the discussed process is very challenging due to the danger of overheating. Conversely, good-quality optical layers deposited on flexible substrates would open up a new perspective for applications in “green” organic electronics and photovoltaics [27]. Typically, the flexible substrates of practical importance are transparent polymeric sheets. Therefore, different improvements and the careful adjustment of the experimental parameters are necessary to successfully use sputtering-based techniques.

Fortunately, thin TiO_2_ layers can be effectively deposited in a controlled manner using a range of well-established wet techniques. In contrast to the vacuum-related methods, wet techniques are usually carried out at the ambient temperature. Of particular interest is the sol–gel procedure [28], which allows extended control over the structure of the final material. A sol is a stable colloidal suspension that collapses under varied thermodynamic conditions, leading to the formation of a gel—a porous three-dimensional interconnected solid network. Depending on the processing details, bulk material, powders or layers can be obtained. Furthermore, the sol–gel technique is straightforward and does not require any sophisticated equipment.

The spin-coating or dip-coating technique is usually used to deposit a sol in a layer form. Both techniques are widely employed in laboratory practice. However, the thickness of the layers obtained by spin-coating is not uniform and depends on the distance from the axis of rotation. The dip-coating technique has the advantage over spin-coating such that very homogeneous layers can be manufactured [29]. Furthermore, the dip-coating technique, unlike the spin-coating technique, can be upgraded to the industrial scale and continuous layers can be deposited. This technology does not produce environmentally hazardous by-products, and, as it is a low-temperature process, little energy is required. Therefore, this technology can be considered as “green”. The latter reason has led to an increasing focus in recent years on improving sol–gel-made TiO_2_ thin films to further enhance their cost-effectiveness and high optical quality [30]. Initially, as deposited sol–gel TiO_2_ thin layers are amorphous, not fully condensed and contain large amounts of organic substances, the transformation of these amorphous gels into a rigid crystalline TiO_2_ material requires subsequent thermal annealing (calcinations).

TiO_2_ thin layers thermally annealed at 300 °C still remain amorphous [31]. If this process is prolonged, a decrease in the layer thickness is observed. This phenomenon can be explained in such a class of materials by the removal of the residual solvent and the collapse of the existing voids [32,33]. Characteristic anatase X-ray diffraction peaks appear in the vicinity of 400 °C [31]; however, this transition, assessed by differential thermal analysis (DTA), occurs at a slightly higher temperature [31]. The transformation of anatase to rutile proceeds at a measurable rate at no less than 600–700 °C [34].

Although the refractive index of TiO_2_ in the amorphous form is already high, its value can be further increased by converting the material to anatase or rutile, which requires elevated temperatures. However, in the case of flexible polymer substrates, only so-called performance polymers can withstand long-term temperatures higher than approximately 200 °C. These are mainly polyimide derivatives [35] headed by poly-oxydiphenylene-pyromellitimide (DuPont trade name—Kapton) that remain stable at 400 °C and survive impact heating at even higher temperatures [36]. Thus, it is possible to determine the maximum temperature of the conversion of amorphous TiO_2_ on such substrates to not more than 600 °C. Therefore, it will certainly not be possible to obtain a layer containing rutile, but the choices in temperature and annealing time enable optimizing the optical properties. On the other hand, a thin layer of sol deposited on the substrate is subject to spatial restrictions. The normal and in-plane directions are distinctly different with respect to the different possible surface-related phenomena. It may therefore happen that, as a result of the sol–gel conversion, the refractive index of the layer becomes uniaxially anisotropic. It is well-established that crystals of both anatase and rutile are optically anisotropic [10]. However, it can be assumed that, in the case of a polycrystalline sample, such a feature should cancel out. To confirm the latter hypothesis (what we supposed to be a novelty), a model series of sol–gel samples were prepared on silicon substrates and then thermally annealed, each at a different temperature. Silicon substrates, unlike polymers, are characterized by very precisely defined dispersion relations of refractive and extinction coefficients, which is essential for accurate measurements by spectroscopic ellipsometry (SE). It can be assumed that the obtained results will also be valid for the layers deposited on polyimides since the surface can be easily functionalized to make it compatible with TiO_2_ [37]. Subsequently, a complementary X-ray diffraction (XRD) study was performed. The results were analyzed and compared to standard tabulated data.

Another novelty of the research described in this article also needs to be highlighted. TiO_2_ layers are crucial for anti-reflective coating or dielectric mirrors due to their high refractive indices. For double-layer anti-reflective structures on silicon substrates (Si/TiO_2_/SiO_2_), layers with thicknesses of about 60 nm are optimal [38]. Similar thicknesses are required for the TiO_2_ layers in dielectric mirrors [39]. In contrast, TiO_2_ layers with thicknesses above 100 nm are required for anti-reflective coatings on glass substrates. Such thicknesses could be obtained by applying thinner TiO_2_ layers twice. However, this implies a higher cost to produce a thicker TiO_2_ layer. For this reason, we decided to develop TiO_2_ layers with thicknesses above 100 nm to be fabricated by a single dip-coating process. In earlier studies, we used TiO_2_ tetrabutylorthotitanate Ti(OC_4_H_9_)_4_ (Ti(OBu)_4_) as a TiO_2_ precursor, which other researchers also frequently use. In contrast, in the studies described in this thesis, we used titania (IV) ethoxide Ti(OC_2_H_5_)_4_ (TET) as the TiO_2_ precursor. In the sol–gel method, the most commonly used precursor for TiO_2_ films is Ti(OBu)_4_, whereas TET was not used to produce such thick films. The novelty element of the work is therefore the use of TET as a precursor for the TiO_2_ films produced by the sol–gel method and the demonstration that very good-quality films can be produced using it.

It should be emphasized that, for many incremental studies, such as those reported in this article, it is still necessary to predict and fully control the properties of the TiO_2_ layers derived from the sol–gel before their use on an industrial scale.

## 2. Materials and Methods

### 2.1. Sol Preparation

TiO_2_ layers were prepared by the sol–gel method and dip-coating technique. The TiO_2_ sol was synthesized with titania (IV) ethoxide (TET, p.). TET was mixed with absolute ethanol (EtOH; 99.8%, p.a.) and deionized water, which is necessary for hydrolysis. Hydrochloric acid (HCl, 36%, p.a.) and polyethylene glycol (PEG 300) were used as catalyst and surfactant, respectively. The molar ratios of reagents were TET:EtOH:H_2_O:HCl:PEG = 1:7.07:1.57:0.43:0.14. The reaction was carried out at 50 °C for 2.5 h.

This reaction is very efficient and reproducible. The resulting product (after removal of residual solvent, etc.) consists of titanium and oxygen in virtually the same stoichiometric ratio as in TiO_2_. Our previous tests, carried out by different chemical analysis methods, as well as literature data confirm this fact. Examples are the EDXS analysis results reported in [40,41] (sol–gel films produced by dip-coating), [42] (sol–gel films deposited by spin-coatings) or [43] (powders obtained through sol–gel reaction).

### 2.2. Sample Preparation

**Figure 1:**
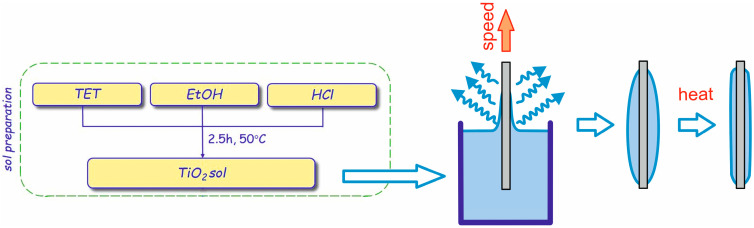
A diagram illustrating the process of obtaining TiO_2_ layers.

TiO_2_ layers were deposited on polish silicon wafers with native oxide. The latter is a kind of surface of very well-defined optical constant, a crucial feature for accurate modelling optical properties of the layer under investigation. Silicon substrate dipped vertically in sol and withdrawn with speed of 5.4 cm/min. After deposition by dip-coating technique, the layers were annealed at 200 °C, 300 °C, 400 °C, 500 °C and 600 °C for 1 h at ambient conditions, as schematically illustrated in Figure 1.

### 2.3. Measurements Using Spectroscopic Ellipsometry

All measurements were carried out using spectroscopic ellipsometer M-2000D from J.A. Wollam, Lincoln, NE, USA. This instrument simultaneously collects data at 709 wavelengths spanning from 1690 nm to 193 nm (0.73 eV to 6.52 eV). The beam of the probing light, approximately 3 mm in diameter, was directed at the sample surface at consecutively increasing angles of incidence near the Brewster’s angle, i.e., (60°, 65°, 70° and 75°). The measurements were carried out in the center of the sample and at six equally distant points surrounding the center within a radius of 5 mm.

The polarization of incident light changes as a result of reflection from the surface, which can be quantified using two so-called ellipsometric angles ψ and Δ [44]. Furthermore, the imperfections of the reflecting surface, as well as the quality of the optical layers on it, contribute to the depolarization of reflected light. The exhaustive quantitative definition of the light with respect to its intensity and polarization (depolarization) can be completed in form of four-term so-called Stokes vector $\left[S_{i}\right]$. This vector consists of the combined intensities of the light beam, occurring as a result of its linear or circular polarization [45].

Two Stokes vectors representing the incoming and the reflected beams can be mutually related by a 4 × 4 matrix, referenced as Mueller matrix, $\left[M_{xx}\right]$. The $M_{11}$ term of this matrix describes the reflected intensity for unpolarized light and conventionally is used to normalize all the other terms, denoted in this case by lower-case letter “m” [46]. After such a normalization, the range of possible values of “m” terms are constrained between −1 and 1. The normalized related Mueller matrix is represented in (1)

(1) $${\left[\begin{array}{c} \begin{array}{c} S_{0}^{\prime }\\ S_{1}^{\prime } \end{array}\\ \begin{array}{c} S_{2}^{\prime }\\ S_{3}^{\prime } \end{array} \end{array}\right]}_{out}=M_{11}\left[\begin{array}{cc} \begin{array}{cc} 1 & m_{12}\\ m_{21} & m_{22} \end{array} & \begin{array}{cc} m_{13} & m_{14}\\ m_{23} & m_{24} \end{array}\\ \begin{array}{cc} m_{31} & m_{32}\\ m_{41} & m_{42} \end{array} & \begin{array}{cc} m_{33} & m_{34}\\ m_{43} & m_{44} \end{array} \end{array}\right]{\left[\begin{array}{c} \begin{array}{c} S_{0}\\ S_{1} \end{array}\\ \begin{array}{c} S_{2}\\ S_{3} \end{array} \end{array}\right]}_{in}$$

The optical configuration of the instruments used in this research was the following: light source–polarizer–rotating compensator–sample–analyzer–detector. This arrangement allows not only measurements of ellipsometric angles ψ and Δ but provides opportunity to calculate 12 out of 16 elements (three upper rows) of the full Mueller matrix. It can be proved that such a matrix of an ideally isotropic surface (with respect to complex refractive index) assumes the simpler form shown in (2) [45,46]:

(2) $$\left[\begin{array}{cc} \begin{array}{cc} 1 & -N\\ N & 1 \end{array} & \begin{array}{cc} 0 & 0\\ 0 & 0 \end{array}\\ \begin{array}{cc} 0 & 0\\ 0 & 0 \end{array} & \begin{array}{cc} C & S\\ -S & C \end{array} \end{array}\right]$$

where N=cos(2ψ), C=sin(2ψ)cos(Δ) and S=sin(2ψ)sin(Δ). In the case of an optically anisotropic matrix, elements $m_{13}$, $m_{14}$, $m_{23}$, $m_{24}$ and $m_{31}$, $m_{32}$, $m_{41}$ and $m_{42}$ are no longer zeroed. To rule out a coincident zeroing, it is enough to rotate the sample (which is flat) around the normal direction and repeat the measurement. In the case of a truly optically isotropic sample, the general structure of the matrix (as shown in (2)) should be insensitive to such rotations. In the reported study, each sample was measured four times and rotated with respect to the initial position by 0, 30, 60 and 90 degrees. The rotation by 90° replaces rows by columns in $\left[m_{ij}\right]$ normalized Mueller matrix, thus providing values of lacking $m_{41}$, $m_{42}$ and $m_{43}$ elements.

Data obtained by SE were analyzed using proprietary software CompleteEase v.6.70, provided by the manufacturer of the instrument J.A. Wollam.

### 2.4. X-ray Diffraction Studies

X-ray diffraction (XRD) is a standard technique to determine (if present) crystalline structure, lattice parameters and size of crystallites. TiO_2_ samples were investigated using an XRD spectrometer (PANalytical X’Pert PRO, Almelo, The Netherlands). A parallel beam of CuKα line (λ = 1.540 Å, 45 kV and 40 mA) and a standard Xe point detector were employed. Data were recorded in the range of 15–80° of 2θ, at intervals of 0.05°, at the scan rate of 0.001°/min. All measurements were carried out in the grazing incidence X-ray diffraction geometry, a version of X-ray diffraction, particularly useful for the study of thin films and coatings. XRD incident beam angle was set to 1.095°.

### 2.5. Surface Imaging by Scanning Electron Microscopy (SEM)

Images of the manufactured surfaces were obtained by means of scanning electron microscopy, using the SEM Supra 35 (Zeiss, Jena, Germany) in the in-lens mode with accelerating voltages ranging from 2 to 10 kV.

## 3. Results and Discussion

**Figure 2:**
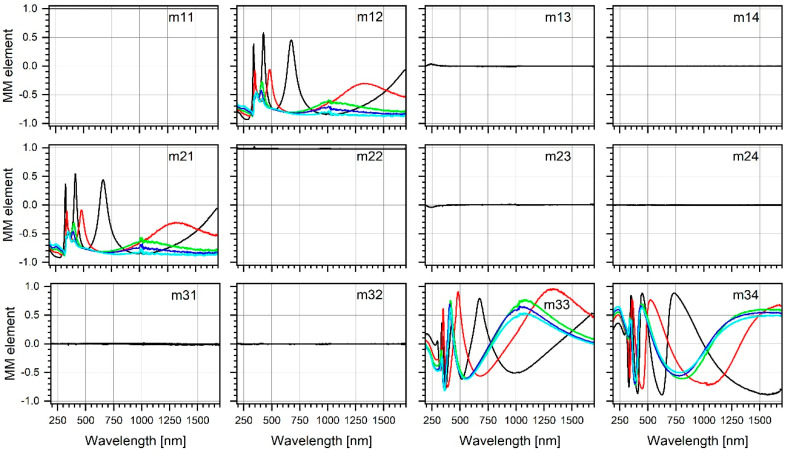
Wavelength dispersion of normalized Mueller matrix elements of the samples annealed at T = 200 °C (black line), T = 300 °C (red line), T = 400 °C (green line), T = 500 °C (blue line) and T = 600 °C (cyan line). The measurements were conducted at the angle of incident light equal to 65°.

In the preliminary analysis, the focus was on the spectra of the normalized elements of the Mueller matrix measured for all the experimental points and incidence angles of the probing light. This approach enables in particular to determine the isotropy (or lack thereof) of the optical properties of the examined TiO_2_ layers. To avoid unnecessarily increasing the volume of text, only one selected example result is shown in Figure 2. The drawing consists of graphs illustrating the wavelength relationship of some matrix elements (12 of 16, i.e., the top three rows). The elements $m_{13}$, $m_{14}$, $m_{23}$, $m_{24}$, $m_{31}$ and $m_{32}$ identically equal zero, regardless of the wavelength. The results of a similar character were obtained for all the other measurements, also for the samples rotated relative to the initial position. In the case of a sample rotated by 90° around the normal direction, the rows in the Mueller matrix are swapped with the columns. Thus, the missing elements of the original matrix, namely $m_{41}$, $m_{42}$ and $m_{43}$, are represented by the elements $m_{14}$, $m_{24}$ and $m_{34}$ in this new matrix [47]. However, regardless of the sample orientation, matrix elements $m_{13}$, $m_{14}$, $m_{23}$, $m_{24}$, $m_{31}$ and $m_{32}$ remained unchanged and equal to zero. Finally, only the $m_{44}$ element remained unknown. As has already been pointed out in the Introduction, the zeroing of $m_{13}$, $m_{14}$, $m_{23}$ and $m_{24}$ and $m_{31}$, $m_{32}$, $m_{41}$ and $m_{42}$ is indirect evidence of optical isotropy [45]. Taking the latter into account, it can be concluded that anisotropy in the optical properties did not appear at any stage of the sample calcination.

This property stands in contrast to that observed for some other types of TiO_2_ thin layers. The ellipsometric studies of epitaxially grown TiO_2_ layers confirmed that the material was essentially oriented with the axis perpendicular to the substrate, and the resulting birefringence was detected [48]. Other studies showed that oblique-angle physical vapor deposition (PVD) can be used to deliberately deposit TiO_2_ thin layers that exhibit in-plane birefringence [49,50]. In the latter case, it was also found that thermal annealing increased the birefringence magnitude.

Spectroscopic ellipsometry does not provide the direct dispersion relations of the refractive index and extinction coefficient. These parameters need to be deduced from the measured values of the ellipsometric angles. To carry out the necessary calculations, the number and nature of the optical layers constituting the sample (the model) have to be assumed. However, different model structures can be proposed. To select the most likely one, a justifying criterion is required. For this purpose, the used software adopts the value of the so-called MSE parameter (acronym for Mean Squared Error), shown in (3).

(3) $$MSE=\sqrt{\frac{1}{3n-m}\sum_{i=1}^{n}\left[{\left(\frac{N_{exp}-N_{fit}}{0.001}\right)}^{2}+{\left(\frac{C_{exp}-C_{fit}}{0.001}\right)}^{2}{+\left(\frac{S_{exp}-S_{fit}}{0.001}\right)}^{2}\right]}$$

where the symbols mean n—the number of wavelengths, m—the number of fit parameters, N=cos(2Ψ), C=sin(2Ψ)cos(Δ) and S=sin(2Ψ)sin(Δ). The best agreement between the modeled data and experimental data is achieved by MSE minimization. A general selection rule states that the ideal model should be as simple as possible, but more complicated models cannot yield a much lower MSE value.

As the starting point for fitting ellipsometric data, a simple model was chosen consisting of a Si substrate with a 1.5 nm layer native oxide (determined independently) covered with a homogenous TiO_2_ layer. The surface roughness was estimated using the Bruggeman effective medium approximation [51] presuming 50% TiO_2_ and 50% voids.

Since the absorption edge of TiO_2_ is located at approximately 3.2 eV (approximately 390 nm), the material can be considered transparent in the longer wavelengths and the data can be analyzed using the empirical Sellmeier formula [52]. All the available data for each sample (measured at different incidence angles and different places on the surface) were fitted as a single set, leaving only thickness as the free parameter. The results are collected in Table 1, from which the systematic evolution of the layers due to the annealing process is apparent. Between 200 °C and 400 °C, the thickness of the samples falls rapidly, while their refractive index follows an opposite trend. Both observations, in addition to the removal of the residual solvent, most likely indicate the collapse of the voids in the samples. The existence of empty spaces (voids or pores) inside TiO_2_ has been demonstrated in many reports by direct SEM imaging, for example, in [40]. In one of our previous articles, we concluded the same indirectly [33].

**Figure 3:**
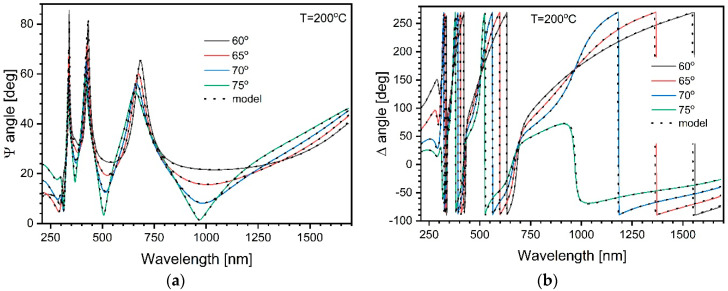
An example (sample annealed at 200 °C) of ellipsometric angles measured using different incidence angles (solid lines) of the probing light beam for (**a**) ψ angle and (**b**) Δ angle and respective B-Spline fits (dotted lines).

These Sellmeier fits were converted to B-Spline polynomials, maintaining the sample thickness constant. Further, the wavelength range for the subsequent B-Spline fits was gradually extended towards the absorbing region and finally covered all the available data. The quality of this curve fitting can be assessed by analyzing, even visually, the graphs in Figure 3.

Attempts to fit more advanced models assuming a refractive index gradient in the normal direction to the sample did not improve the accuracy of the fit. Thus, the obtained results were accepted as truthful. Typically, B-Spline graphs are further deconvoluted into different theoretical line-shapes resulting from the transition to the theoretically assumed bandgap profile [53]. However, such an approach usually requires additional information to provide fully reliable and unambiguous results. Fortunately, for a comparative analysis, this step is not essential.

**Figure 4:**
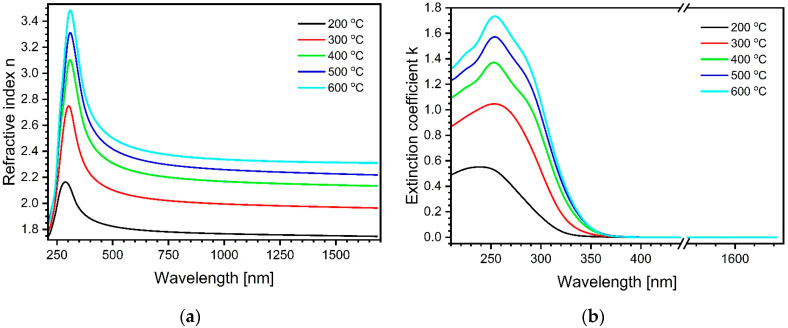
(**a**) Compilation of refractive indices’ dispersion calculated through B-Spline fits for samples annealed at systematically increasing temperatures, as explained in the legend. (**b**) Compilation of extinction coefficients’ dispersion calculated through B-Spline fits for samples annealed at systematically increasing temperatures, as explained in the legend. The break in the abscissa was established to show more clear details of the absorption region.

The derived dispersion relations of refractive index n and extinction coefficient k (shown in Figure 4a and Figure 4b, respectively) are physically correct because the Kramers–Kronig consistency and positive imaginary part of the dielectric constant were imposed during the B-Spline fitting procedure.

From the data presented in Table 1, it can be observed that the thickness decreases as the result of thermal annealing, but the rapid change ends at 400 °C. This observation can be related to the conversion of amorphous TiO_2_ in anatase form. However, some authors [54,55] locate this phenomenon at slightly higher temperatures according to studies on thermogravimetry and differential scanning analysis. On the other hand, such transformations depend very strongly on their kinetics and are spanned over time. The latter is shorter the higher the temperature.

A comparison between the curves in Figure 4a,b for annealing temperatures 200 °C, 300 °C and 400 °C reveals rapidly increasing intensity combined with shape evolution. In contrast, if the annealing temperature equals or exceeds 400 °C, the overall shape of the curves stabilizes and the intensity rises more slowly. Such an impact of the temperature on the level of the refractive index can most likely be explained by the release of volatile solvent residues and then the collapse of the resulting pores in the TiO_2_ [33,54,56]. The phenomenon of the roughness increasing with the annealing temperature, as shown in Table 1, is in accordance with the direct observations of similar surfaces by atomic force microscopy (AFM) [55].

The creation of homogenous anatase from amorphous TiO_2_ can be assessed more quantitatively by the calculation of the bandgap energy. The indirect and direct optical absorption bandgaps of the anatase layer are estimated at approximately 3.2 eV and 3.8 eV, respectively [57,58,59,60,61]. The most common method determines this energy by plotting ${\left(\alpha h\nu \right)}^{1/m}$ versus photon energy (hν), where α stands for the optical absorption coefficient and 1⁄*m* equals 1/2 or 2 for indirect and direct transitions, respectively. Such graphs are referred to as Tauc plots.

Absorption coefficient measurements by classical spectroscopy (no matter whether in transmission or reflection mode) require the use of standards. For solid samples, it is often difficult to find standards that are fully adequate in terms of reflectance and scattering. Consequently, in many articles, the authors include absorption (or reflectance) spectra of TiO_2_, which take a non-physical course. This issue was discussed in detail in [62].

Ellipsometry, in principle, does not require standards, hence its greater reliability in determining the absorption coefficients of solid samples. The absorption coefficient can be easily determined from dispersion relations as α=4πk/λ.

**Figure 5:**
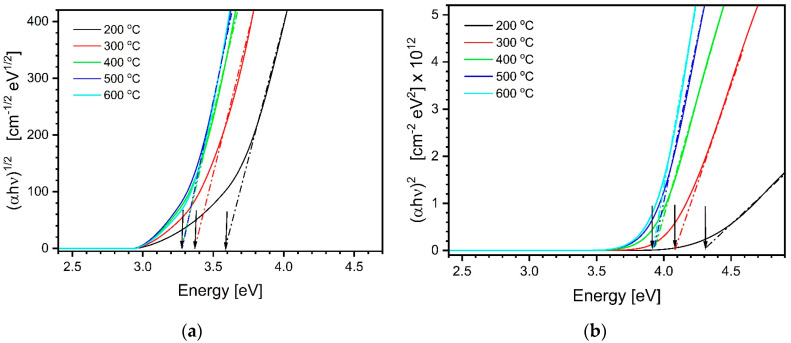
(**a**) Tauc plots drawn to determine in studied TiO_2_ layers indirect bandgap energies. (**b**) Tauc plots drawn to determine in studied TiO_2_ layers direct bandgap energies. The mentioned energies are indicated at abscissa by arrows.

The Tauc plots obtained from the data shown in Figure 4a are illustrated in Figure 4b and Figure 5a. Alternatively, one can calculate the bandgap energy using the Tauc–Lorentz model oscillator [63]. It depicts the absorption of amorphous materials as a broadened Lorentzian line with zero absorption below the bandgap and uses dielectric function formalism. The formula in (4) describes the imaginary part of the complex dielectric function resulting from this model oscillator, where $E_{0}$ stands for the center energy of the resonance, $E_{g}$ is the bandgap energy, $B_{r}$ is the line broadening, and A is the amplitude (all parameters in eV). The real part of the complex dielectric function can be calculated through Kramers–Kronig transformation.

(4) $$\varepsilon_{2}\left(E\right)=\frac{AE_{0}B_{r}{\left(E-E_{g}\right)}^{2}}{{\left(E^{2}-E_{0}^{2}\right)}^{2}+B_{r}^{2}E^{2}}\cdot \frac{1}{E}\;\;\;if\;E\geq E_{g}\;,\;\;\varepsilon_{2}\left(E\right)=0\;\;if\;\;\;E<E_{g}$$

**Figure 6:**
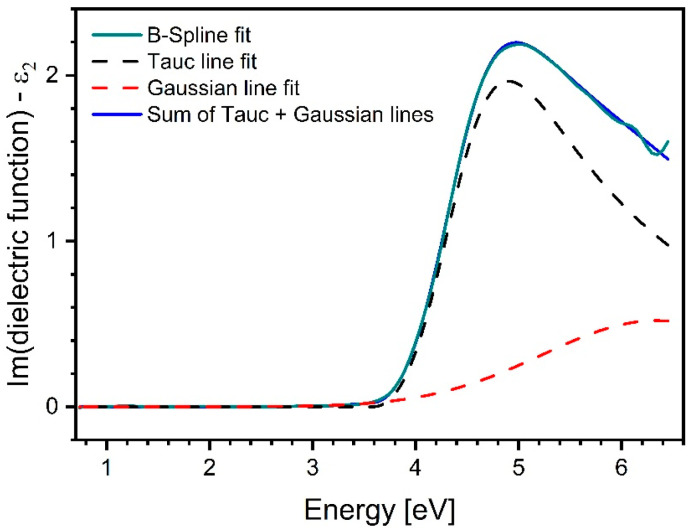
Imaginary part of complex dielectric function resulting from Tauc–Lorentz oscillator model for the sample annealed at 200 °C. Necessary data were adapted from Figure 3a,b.

Clearly, visible maxima were hardly possible to determine in the dispersion of $\varepsilon_{2}$ derived from the available experimental data (refractive indices and extinction coefficients, as shown in Figure 4a,b). In practice, absorption bands extend outside the available wavelength range, and different bands often converge to a point at which they are not possible to be separated unambiguously. This fact severely restricts the accuracy of theoretical model fitting. Taking into account the latter, only one Tauc–Lorentz oscillator was fitted exclusively to the rising slope of the curve, just above the absorption edge, while the remaining part was approximated as the falling arm of a Gaussian line. The sample thickness included in the fit was taken from Table 1 and kept constant. An example of such treatment of the experimental data is shown in Figure 6, and only the lower bandgap energy was determined.

The fitted bandgap energies are summarized in Table 2. Although the values found using the Tauc–Lorentz model are slightly larger, the overall trend of the dependence on the temperatures similar. In contrast to the samples annealed at 200 °C and 300 °C, the energies calculated for samples annealed at 400 °C and above are constant in value as if the electronic structure of the material reached its final state.

**Figure 7:**
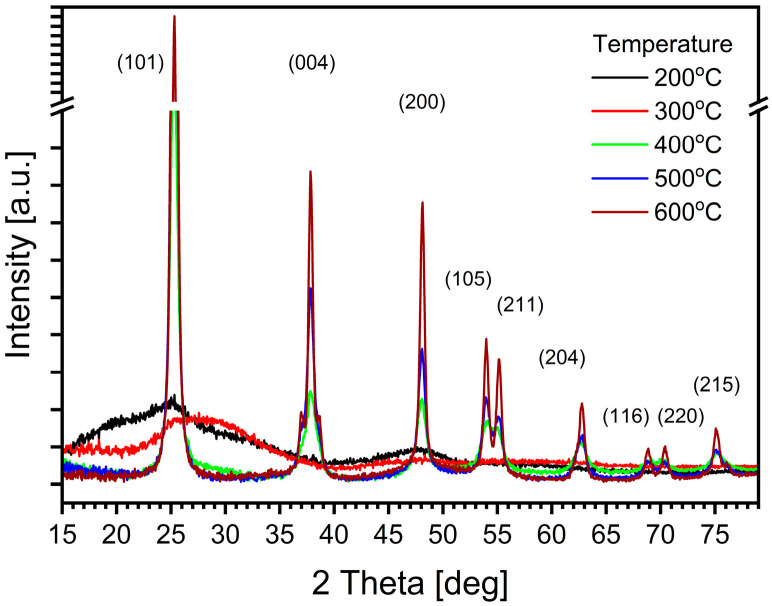
Comparison of XRD diffractograms recorded for samples annealed at different temperatures. The first main peak of the anatase phase, much stronger than the others, appears at 25.33°2θ. To better visualize weaker peaks, the vertical axis is divided and contains a gap.

Complementary information on the investigated TiO_2_ layers was obtained by XRD. In Figure 7, the XRD diffractograms recorded for all the investigated TiO_2_ samples are summarized. Only very broad maxima, typical of the amorphous phase, appeared for the samples annealed at 200 °C and at 300 °C, too low a temperature to activate the crystallization process. Well-defined, sharp 2θ diffraction peaks were observed exclusively for the samples annealed at 400 °C or above. This observation confirms the latter temperature being the threshold for the conversion of amorphous TiO_2_ into a crystalline form.

The diffraction peaks appeared at 2θ equal to 25.28° (the strongest one), [36.01°,37.90°], 48.06°, 53.96°, 55.06°, 62.70°, 69.01° and 70.41°. These values, according to JCPDS standard card No. 84-1285, are characteristic of the anatase phase, with respect to the following planes—(101), [(103), (004), (112)], (200), (105), (211), (204), (116) and (220). In other words, the observed TiO_2_ crystallites had a truly tetragonal anatase structure with trigonal planar and octahedral geometry [64,65]. The recorded XRD diffractograms did not have any additional features characteristic of rutile or any other crystalline structure (including potentially present impurities).

The intensity of the observed peaks increased with the annealing temperature to which the samples were subjected. The broadening of these peaks was used to calculate the average crystalline size D (coherence length) of the TiO_2_ crystallites according to the Debye–Scherrer formula, as shown in (5).

(5) $$D=\frac{K\lambda }{\beta cos\theta }$$

where K is a dimensionless Sherrer constant, λ is the wavelength of X-rays and β is FWHM (full width at half maximum).

The results collected in Table 3 evidence that the average size of the crystallites increased with the annealing temperature, more than twice, from 11 to 24 nm. Comparing the relevant values in Table 2 and Table 3, it can be seen that the size of the crystallites has no effect on the absorption edge.

The values of the crystallite sizes were confirmed by the Williamson–Hall plot method [66]. Also using the latter method, lattice strain was determined as the slope of βcos⁡θ plotted against 4sin⁡θ.

The dislocation densities were calculated using (6) [40,67]

(6) $$\delta =\frac{1}{D^{2}}$$

The probability of a stacking fault (planar defect) that results in slight shifts in the diffraction peaks was estimated using (7) [67].

(7) $$\alpha =\beta \;\frac{2\pi^{2}}{45\sqrt{3\tan\theta }}$$

The resulting further set of structural parameters deduced from the XRD patterns are shown in Table 4.

Dislocation is a parameter describing irregularities in the crystal structure. From the results obtained, it is clear that the dislocation density decreases with increasing temperature. At lower annealing temperatures, the atoms at the surface have lower energy and the surface mobility is low. This results in the appearance of more defects. As the temperature increases, the atoms gain enough kinetic energy and surface mobility for crystal growth and occupy stable positions in the crystal structure. The number of dislocations decreases. Similarly, the lattice strain decreases with increasing temperature, indicating fewer lattice dislocations in growing crystallites of larger sizes. As the size of the crystallites increases, the surface-to-volume ratio decreases, resulting in fewer atoms near the surface of the crystallites. At the same time, the number of atoms in the whole crystallite increases, which means that the structure of the crystal lattice becomes more ordered and less distorted. These two effects tend to reduce the amount of stress in the system.

In the diffractograms, only slight shifts in the position and changes in the intensity of the diffraction peaks can be observed as a function of the increasing annealing temperature. Assuming that the crystalline structure is anatase, one can determine the changes in lattice constants a, b and c of the elementary cell (as shown in Table 4). Their values decrease slightly, approaching those of the relaxed reference powder samples. The calculated stacking defect probability α (the fraction of layers that undergo sequential stacking defects) shows a similar trend. Both effects mentioned above are evidence of the improved structural ordering under the influence of higher annealing temperatures.

**Figure 8:**
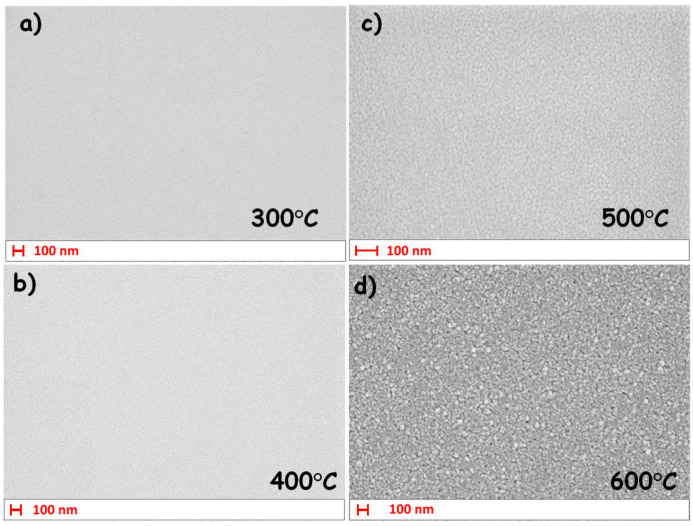
Comparison of SEM images of samples annealed at different temperatures (**a**) 300 °C, (**b**) 400 °C, (**c**) 500 °C, (**d**) 600 °C. The horizontal bar is the scale of the photograph.

Another interesting observation is the correlation between the crystallite size confirmed by XRD (shown in Table 3) and the surface roughness found by spectroscopic ellipsometry (shown in Table 1). The surface of the amorphous layer is the smoothest and the roughness of the rest increases with the size of the crystals. The most natural explanation is that, in the case of polycrystalline material, nanocrystals can partially protrude above the surface of the layer. Figure 8 shows the results of the SEM observations that confirmed this obvious hypothesis.

From a comparison of the results shown in Table 3 and Table 4, it can be seen that the increased annealing temperature causes crystal growth and a concomitant decrease in the number of defects. The material thus becomes more homogeneous and therefore potentially achieves lower losses. On the other hand, as can be seen from Table 1 and Figure 8, the surface of the layer becomes rougher. This is particularly undesirable for single-mode waveguide layers with thicknesses slightly above the fundamental mode cut-off thickness, where the roughness of their surface is the dominant source of loss [68]. Consequently, the technological challenge to minimize the losses in such waveguides is to balance the impact of these two factors.

## 4. Conclusions

A series of TiO_2_ thin films, which are polycrystalline anatase, were investigated. They were obtained by the thermal conversion of the amorphous layers deposited by the sol–gel technique. However, this crystallinity did not involve noticeable optical anisotropy, as evidenced by the ellipsometry measurement in the Mueller matrix formalism. In other words, the refractive index of such layers does not depend on the orientation with respect to the surface. This characteristic persists regardless of the annealing temperature. To the best of our knowledge, this phenomenon has not yet been described anywhere. Nevertheless, the temperature affected the morphological parameters of the layers, such as the surface roughness or crystallite size. It is intriguing that crystallites reaching a size of a few percent of the layer thickness do not cause macroscopic birefringence. Apparently, they are arranged in a completely statistical manner despite the boundary of the medium in close proximity.

Undoubtedly, the efficient conversion to anatase occurs at 400 °C, a temperature that efficient polymers can withstand. This fact opens up the prospect of efficiently producing high-quality optical waveguides on flexible substrates.

## Figures and Tables

**Table 1:** Values of selected parameters derived from Sellmeier fit. The thickness is averaged over seven mapped points (as explained in Materials and Methods section).

Sample Annealing Temperature [°C]	Thickness [nm]	Roughness [nm]	Refractive Index at λ = 632 nm
200	323.6	0.89	1.791
300	187.0	1.35	2.039
400	144.1	1.54	2.217
500	124.5	1.76	2.226
600	122.6	2.98	2.318

**Table 2:** Energies of absorption edge in [eV] and equivalent wavelengths in [nm] (in parentheses). (A) indirect transition energy deduced from Figure 4a; (B) direct transition energy deduced from Figure 4b; (C) indirect transition energy fitted using Tauc–Lorentz oscillator model.

Sample Annealing Temperature [°C]	(A) Indirect	(B) Direct	(C) Indirect
200	3.59 (345.4)	4.30 (288.4)	3.57 (347.3)
300	3.37 (368.0)	4.07 (304.7)	3.40 (364.7)
400	3.29 (376.9)	3.94 (314.7)	3.34 (371.3)
500	3.27 (379.2)	3.91 (317.1)	3.35 (370.1)
600	3.28 (378.0)	3.93 (315.5)	3.34 (371.3)

**Table 3:** Crystalline samples—crystallites’ sizes were estimated from the main peak of crystalline anatase (101).

Sample Annealing Temperature [°C]	2*θ* (101) Peak Position [deg]	2*θ* FWHM β [deg]	Intensity [a.u.]	Crystallite Size *D* [nm]
400	25.276	0.831	262	11
500	25.290	0.637	563	15
600	25.314	0.442	1329	24

**Table 4:** The analysis of the lattice parameters, lattice strain, dislocation densities and stacking fault probability of crystalline thin films of TiO_2_ refined from diffraction profiles.

Sample Annealing Temperature [°C]	Lattice Parameters			Lattice Strain [%]	Dislocation Densities δ [1/m^2^]	Stacking Fault Probability α [%]
	a = b [Å]	c [Å]	α = β = γ [deg]			
400	3.7849	9.5880	90	1.60	6.86·10^15^	0.71
500	3.7835	9.5745	90	1.22	3.86·10^15^	0.55
600	3.7797	9.5700	90	0.83	1.71·10^15^	0.35

## Data Availability

The raw data supporting the conclusions of this article will be made available by the authors on request.
